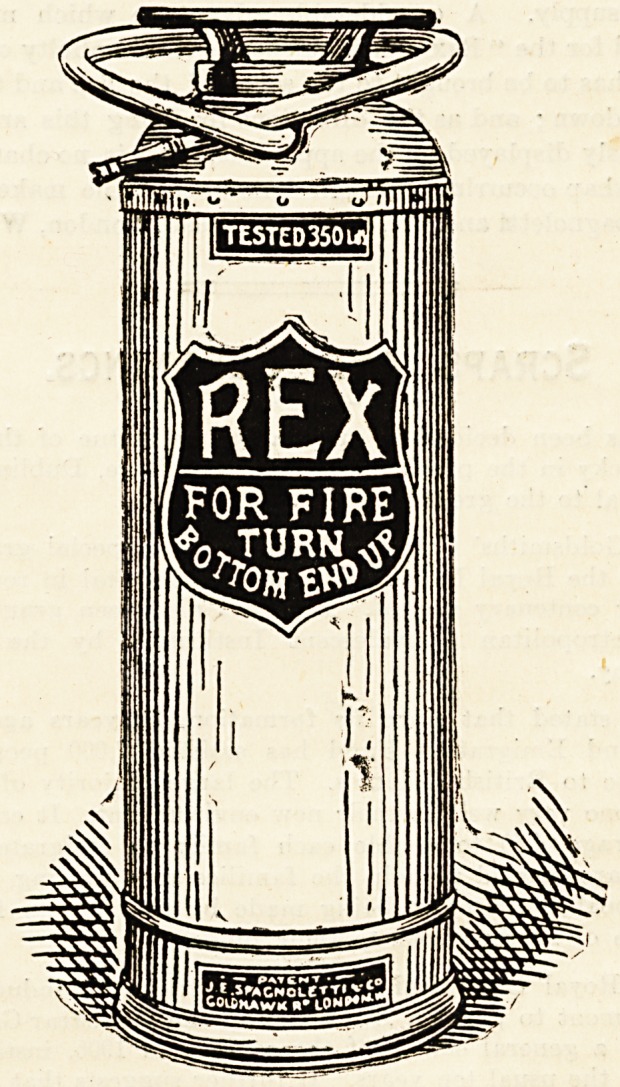# Practical Departments

**Published:** 1904-03-26

**Authors:** 


### PRACTICAL DEPARTMENTS.

### "REX" FIRE EXTINGUISHER.

It is one of the essential duties of the authorities of every large inhabited building, especially where a portion of the inhabitants consists of sick or infirm persons, to exercise sufficient foresight with regard to fire dangers. That this duty is sometimes neglected is only too apparent from the disasters which are from time to time reported in the daily papers. The inevitable criticism which follows such an occurrence usually renders it clear that a little forethought and timely preparation would have averted the catastrophe. Unfortunately, it is often only after some drastic experience, attended perhaps with the loss of human lives, that precautions are taken which should have been provided long before. The natural impulse is to regard water as the best means of defence against fire; but one drawback to this is that a comparatively large quantity is needed to extinguish a small conflagration. Again, the damage done by the large volume of water required may be considerable. Much better protection is provided by one of the patent fire-extinguishers now on the market. We have recently examined a most admirable type of machine which is sold under the name of "Rex" fire extinguisher. It consists of a three-gallon copper cylinder to which is attached an indiarubber tube which acts as a hose. The cylinder is filled with water containing bicarbonate of soda. Immersed in this is a bottle of commercial sulphuric acid which is securely sealed. By an ingenious mechanism the sulphuric acid is allowed to escape into the soda solution when the apparatus is inverted, and the rapid evolution of carbon dioxide causes the mixture to be forced out in the form of a jet which carries a considerable distance. There is a handle at the bottom of the cylinder to facilitate holding the apparatus in the inverted position. As soon as it is empty the instrument can be quickly and easily recharged so as to be ready for further use.

We recently put the "Rex" extinguisher to a pretty severe test. A large fire of wood, soaked with paraffin, was made, and when it had been burning for some little time to let the centre get thoroughly burnt through, a quantity of dry brush wood was thrown on. When this had well caught alight the extinguisher was brought into play, and in a few seconds the flames and smoke were entirely subdued. This was in an open field with a strong wind blowing. It is obvious that in a closed space the result produced would be much greater, since the extinguishing effect is due to the $CO_2$ evolved from the mixture; moreover, a fire such as that with which the test was carried out would probably have been already beyond the control of any domestic water supply. A considerable advantage which may be claimed for the "Rex" extinguisher is its simplicity of use; it only has to be brought to the scene of the fire and turned upside down; and as the directions for doing this are conspicuously displayed on the apparatus, there is no chance of any mishap occurring through ignorance. The makers are J. E. Spagnoletti and Co., Goldhawk Road, London, W.

## Figures and Tables

**Figure f1:**